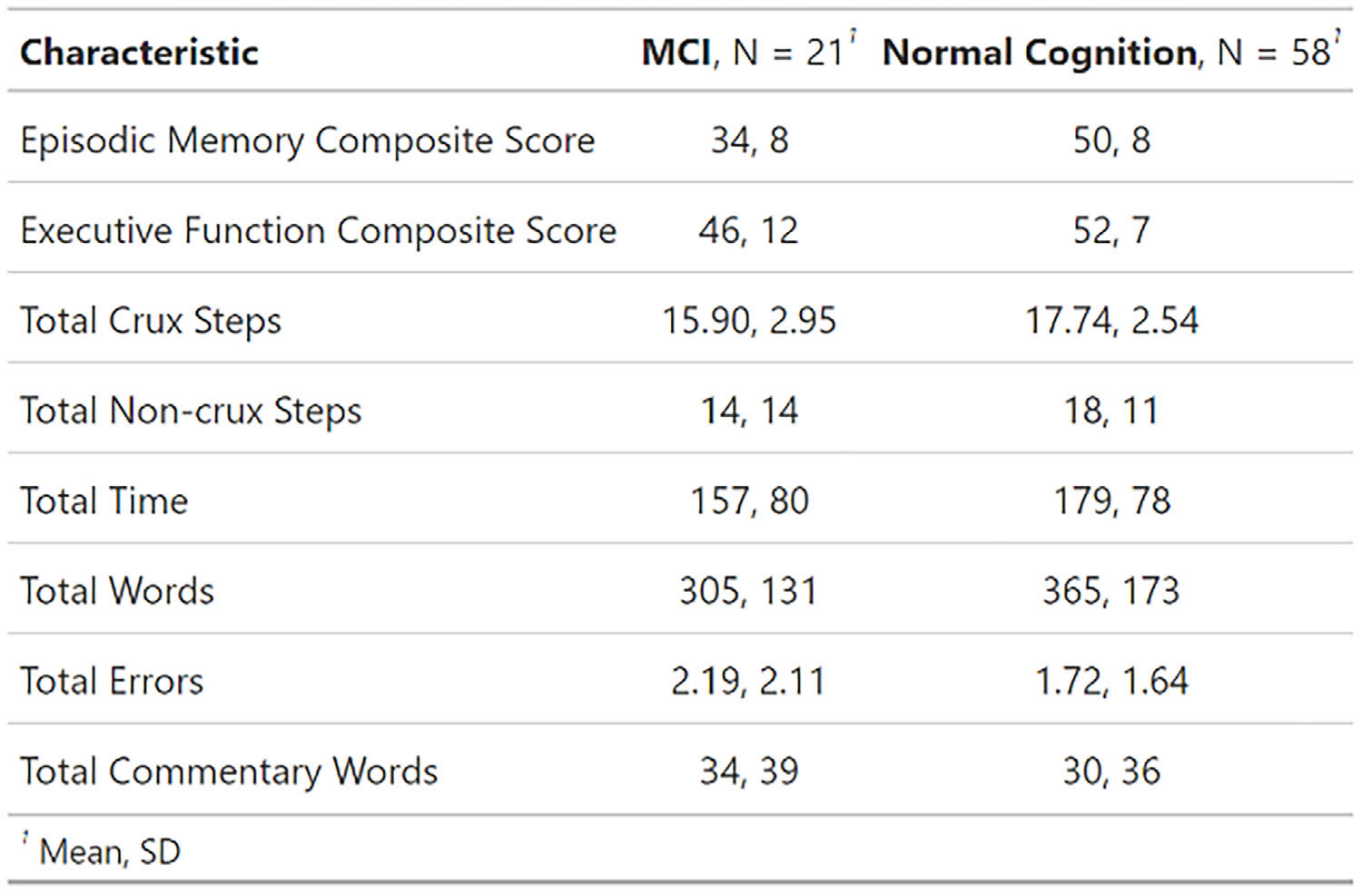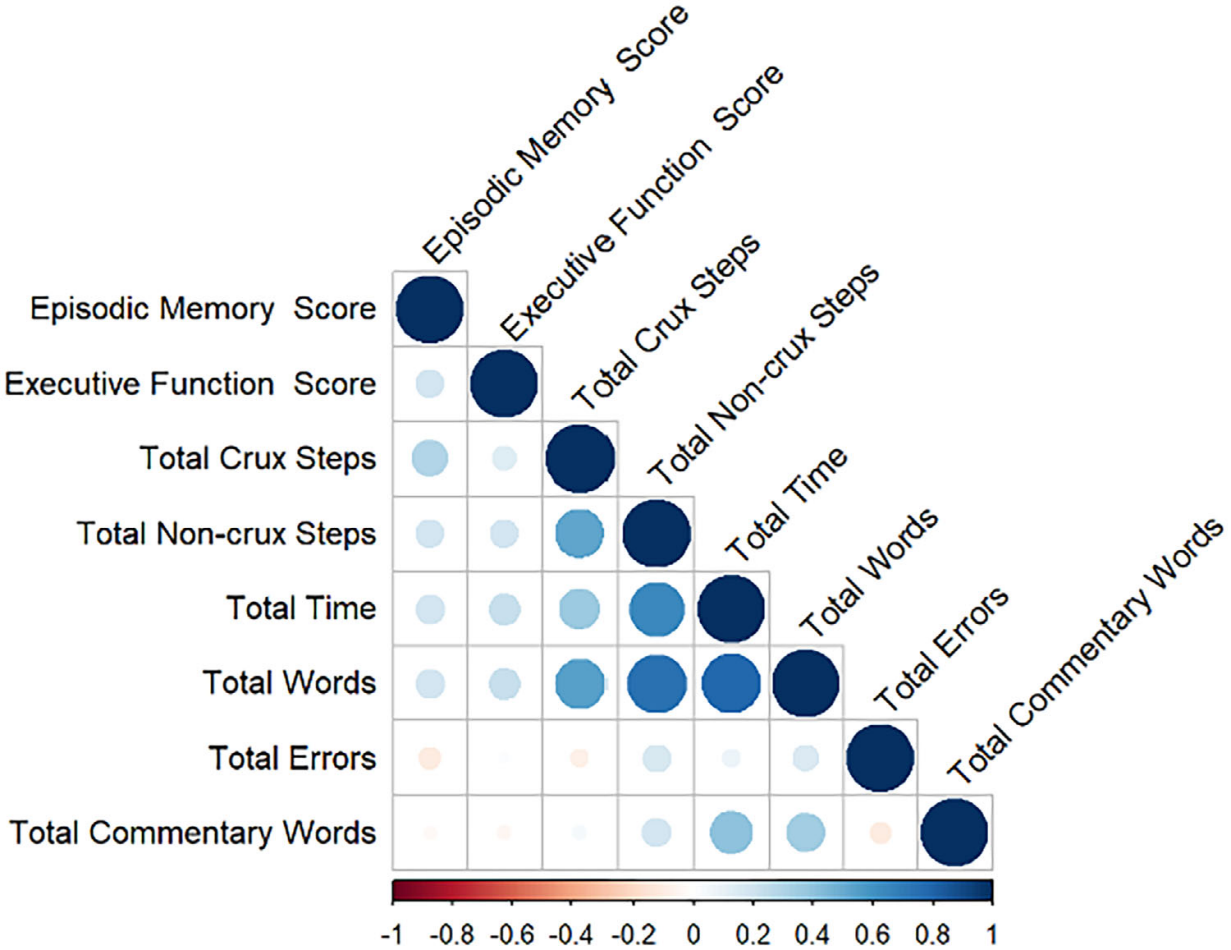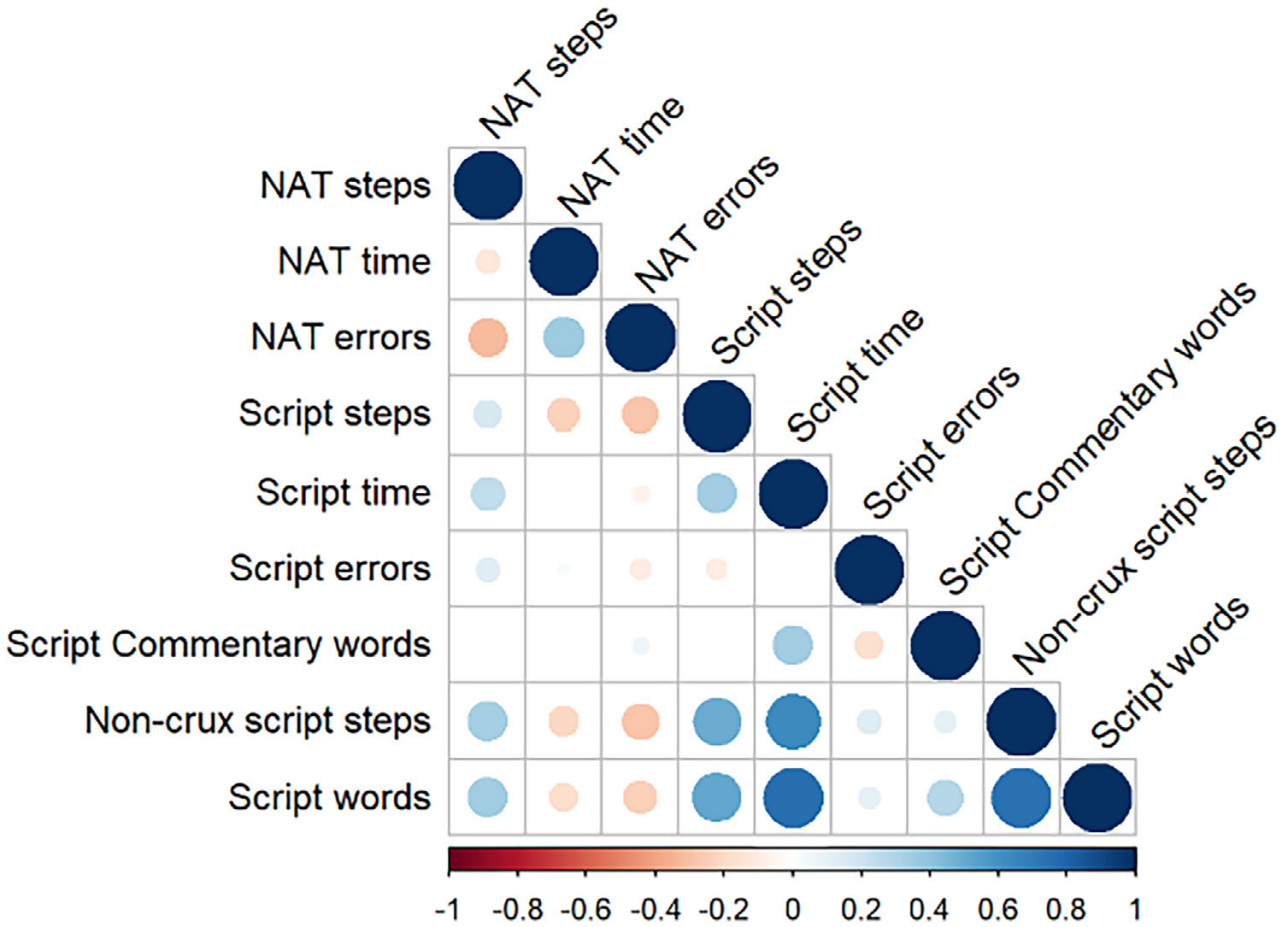# Relations between language production and everyday functioning in older adults

**DOI:** 10.1002/alz.092556

**Published:** 2025-01-03

**Authors:** Melissa Rosahl, Marina Kaplan, Moira Mckniff, Sophia L. Holmqvist, Riya Chaturvedi, Adaeze Uwaomah, Anna Callahan, Daniela McCourt, Emma Pinsky, Giuliana Vallecorsa, Simone Brown, Tania Giovannetti

**Affiliations:** ^1^ Temple University, Philadelphia, PA USA

## Abstract

**Background:**

Subtle changes in naturalistic language have been associated with cognitive abilities in older adults. This study explored script generation as a method for detection of mild cognitive impairment (MCI). Differences in human‐coded speech analysis of scripts were examined between MCI vs. Healthy Controls.

**Method:**

79 older adults aged 65+ (n = 21 MCI, n = 58 normal cognition) completed a verbal script generation task (SGT) requiring descriptions of the following: making toast, coffee, a sandwich, etc. Scripts were scored for total time, number of words, essential and non‐essential steps, errors, and commentary words. Participants also performed neuropsychological testing and a Naturalistic Action Task (NAT), which required making meals with real objects. NAT performance was scored for total time, accomplishment steps, and errors. One‐way ANOVAs were conducted to compare MCI vs. Controls on the SGT. Spearman correlations were conducted to examine relations between the SGT and other measures (neuropsychological tests/NAT).

**Result:**

Participants with MCI generated significantly fewer essential steps on the SGT (Kruskal Wallis Chi‐Squared = 7.61, df = 1, p < 0.001). Correlation analyses showed that participants who generated more essential steps on the SGT had better episodic memory scores (r = 0.29, p <0.01). Participants with better executive function took more time to complete the SGT (r = 0.23, p = 0.04) and generated more words on the SGT (r = 0.23, p = 0.04).

SGT scores were significantly associated with NAT scores. More essential steps on the SGT were associated with fewer NAT errors (r = ‐0.27, p = 0.02). More non‐essential steps were associated with greater accomplishment of NAT steps (r = 0.30, p = 0.01) and fewer NAT errors (r = ‐0.27, p = 0.0216). Longer script time and more script words were associated with greater NAT accomplishment (script time: r = 0.25, p = 0.034). Greater total script words were associated with both greater NAT accomplishment (r = 0.35, p<.01) and fewer NAT errors (r = ‐0.23, p = 0.05).

**Conclusion:**

Script generation scores significantly differentiated participants with MCI versus controls and were associated with independent measures of cognition and performance of everyday tasks. SGTs hold great promise as screening tools for cognitive decline and functional impairment. Automated scoring programs are needed to facilitate the use of script tasks in clinics and large‐scale research.